# Fabrication and Development of Pectin Microsphere of Metformin Hydrochloride

**DOI:** 10.5402/2012/230621

**Published:** 2012-08-01

**Authors:** Pritam Banerjee, Jyotirmoy Deb, Amitava Roy, Amitava Ghosh, Prithviraj Chakraborty

**Affiliations:** ^1^Department of Pharmacy, Himalayan Pharmacy Institute, Majhitar, Rangpo, East Sikkim 737136, India; ^2^Bengal College of Pharmaceutical Sciences and Research, Bidhannagar, West Bengal, Durgapur 713212, India

## Abstract

*Purpose*. The objective of the proposed work is to evaluate the efficacy of Pectins to qualify them as polymers for designing an oral microsphere for the delivery of selected oral antidiabetic drug-like metformin hydrochloride. *Methods*. Different Microspheres formulations were prepared by the water in oil (w\o) emulsion solvent evaporation technique and subsequently evaluated for its different physical parameters as well as its *in vitro* and *in vivo* drug release study. *Results*. The formulations F2 (98.42) and F3 (98.03) showed a constant and high release in the dissolution profile, so among these two formulations, F2 was taken for development study, due to the better result shown over in other evaluation parameters. From the HPLC determinations after *in vivo* study, it had been found that the test samples and the standard sample had not shown any significant fluctuation in relation to their retention time. *Conclusion*. From *in vitro* and *in  vivo* results, it may be concluded that drug-loaded pectin microspheres in 1 : 1 ratio are a suitable delivery system for metformin hydrochloride and may be used for effective management of NIDDM. From this experiment, it could be concluded that as a natural polymer, pectin has potentiality in novel drug delivery system.

## 1. Introduction

The novel system of drug delivery offers a means of improving the therapeutic effectiveness of incorporated drugs by providing sustained, controlled delivery and targeting the drug to desired site. A number of systems containing various types of polymer and wax were fabricated with drugs into dosage form with the aim of sustaining drug levels and hence drug action is obtained for an extended period of time [1]. However, a lack of understandings of anatomical and physiological barriers imposed impediment on the development of efficient delivery system. The modern era of controlled release technology represents the period in which an attempt at drug development is emphasized. The drug delivery system should deliver a drug at a rate dictated by the needs of the body over a specified period of treatment [2].

Pectin was first isolated and described in 1825 by Henri Braconnot, though the action of pectin to make jams and marmalades was known long before. To obtain well-set jams from fruits that had little or only poor quality pectin, pectin-rich fruits or their extracts were mixed into the recipe. During industrialization, the makers of fruit preserves soon turned to producers of apple juice to obtain dried apple pomace that was then cooked to extract pectin [3]. Naturally, pectin in the form of complex, insoluble protopectin is part of the nonwoody parts of terrestrial plants. In the middle lamella between plant cells, pectin helps to bind cells together and regulates water in the plant. These are methylated ester of polygalacturonic acid and it is commercially extracted from citrus peels and apple pomace under mild acidic conditions [4].

Metformin HCl (MFH) is indicated for patients with noninsulin-dependent diabetes mellitus (NIDDM), particularly those with refractory obesity [5].

## 2. Materials and Method

MFH was a kind gift from Zydus Health care (Sikkim, India). Pectin was purchased from Loba Chemie (Mumbai, India). HPMC was purchased from Loba Chemie (Mumbai, India). Ethyl cellulose and Acrycoat S100 were purchased from Sd Fine-chemicals (Mumbai, India) and Corel Pharma (Ahmedabad, India), respectively. All other chemicals used in the study were of analytical grade.

## 3. Methodology

### 3.1. w*\*o Emulsion Solvent Evaporation Method [6]

Microspheres were prepared by the water in oil (w*\*o) emulsion solvent evaporation technique. The drug is dissolved in polymeric aqueous solutions. The solution was poured into 200 mL of paraffin liquid containing 0.5% span 80 as an emulsifying agent. The aqueous phase was emulsified into the oily phase by stirring the system in a 500 mL beaker and its content was heated by a hot plate at 80°C. Stirring and heating was maintained for 2.5 hr till the aqueous phase was completely removed by evaporation. The light oil was decanted and collected microsphere washed three times with 10 mL hexane, filtered through whatmann filter paper, dried in an oven for 2 h, and stored in desiccators at room temperature.

Formulation F1–F8 was prepared by using drug with different ratios of pectin (1:.5, 1 : 1, 1 : 1.5, 1 : 2, and 1 : 2.5, 1 : 3, 1 : 3.5, and 1 : 4) and formulation F9–F12 was prepared by using drug with different polymers in same ratio (1 : 1) for further development.

### 3.2. Percentage Yield [7]

The yield was calculated as the weight of the microspheres recovered from each batch divided by total weight of drug and polymer total amount of that particular batch multiply by 100.

### 3.3. Drug Content Estimation and Drug Entrapment Study [8]

The loaded microspheres (100 mg) were powdered and suspended in 100 mL methanolic water (1 : 99 v*\*v) as a solvent. The resultant dispersion was kept for 20 mins for complete mixing with continuous agitation and filtered through a 0.45 *μ*m membrane filter. The drug content was determined spectrophotometrically (UV-1700, Shimadzu, Japan) at 233 nm using a regression equation from the standard graph.

The drug entrapment efficiency was calculated by the equation

(1) DEE=  (PcTc)×100.

Here, Pc is the practical content, Tc is the theoretical content, and all the experimental unites were analyzed in triplicate.

### 3.4. Percent of Moisture Loss [8]

The MFH loaded microspheres of different polymers were evaluated for percentage of moisture loss which shares an idea about its hydrophilic nature. The microspheres weighed initially and kept in a desiccators containing calcium chloride at 37°C for 24 hours. The final weight was noted when no further change in weight of sample.

### 3.5. Determination of Size Distribution of Microspheres [9]

The microspheres were sized and photographed in normal saline containing 0.1% Tween 80 to prevent aggregation under a light microscope (Olympus C 011, Japan) equipped with an ocular micrometer and a light camera (Seagull DF-1, China). Two hundred microspheres were sized by the above-mentioned method and the mean diameter as well as size distribution of microspheres were determined.

### 3.6. Morphological Study Using Scanning Electron Microscopy (SEM) [10]

The microspheres are studied under photomicroscope RXL-5T (Carton Optical Industries Ltd, Burdwan University, West Bengal, India) and observed for the distribution of drug and polymer in the patches. The surface morphologies of the microspheres were investigated by using scanning electron microscope, samples were gold coated to make them electrically conductive.

### 3.7. Drug Polymer Interaction Study

#### 3.7.1. Fourier Transform Infrared Radiation Measurement (FT-IR) [11]

The FTIR spectral measurements were taken at ambient temperature using IR spectrophotometer (Shimadzu, model 840, Japan). Two mg of pure drug, empty microspheres, and drug-loaded microspheres were selected separately.

#### 3.7.2. *In Vitro* Drug Release [12]

*In vitro* drug release study was carried out in USP XXI basket-type dissolution test apparatus using 6.8 pH phosphate buffers as a dissolution medium. Volume of dissolution medium was 900 mL. Bath temperature was maintained at 37 ± 1°C throughout the study. Basket speed was adjusted to 50 rpm. Samples were withdrawn (5 mL) in 5 min, 10 min, 15 min, 20 min, 30 min, 45 min, 60 min, and then an interval of 1 h. up to 9 h with replacement of 5 mL fresh medium and analyzed for MFH content by UV-visible spectrophotometer at 233 nm. All the experimental units were analyzed in triplicate (*n* = 3). The *in vitro* dissolution data is summarized in Figure 3, for F1 to F8. All the formulations found to release MFH in a controlled manner.

In order to study the exact mechanism of drug release [13] from microspheres, drug release data was analyzed according to zero order, first order [14], and Higuchi's square root [14, 15]. The criterion for selecting the most appropriate model was chosen on the basis of goodness of fit test.

#### 3.7.3. Statistical Analysis [8, 16]

All the results obtained during evaluation were verified with different statistical analyses like one-way ANOVA, standard deviation, and probability log scale plotting (for measurement of particle size).

#### 3.7.4. *In Vivo* Evaluation [17, 18]

Rabbits (New Zealand, white) of either sex weighing (2.8–3.2 Kg) are divided into four groups, each consisting of six animals. First group was taken as diabetic control, second group received placebo, third group received an oral dose of (25 mg) pure metformin hydrochloride, and third group received an oral dose of 25 mg formulated drug.

 Group 1: Diabetic control,
 Group 2: Placebo,
 Group 3: Diabetic rats treated with 25 mg/kg metformin HCl pure drug,
 Group 4: Diabetic rats treated with 25 mg/kg of pectin microsphere,

The drugs are put behind the tongue to avoid their destruction due to biting. Food was withdrawn from the rabbits 12 hrs before drug administration. All rabbits had free access to water throughout the study. The institutional Animal Ethical Committee approved the protocol for this study.

Blood samples were collected from the marginal ear vein of rabbits at 0, 1, 3, and 5 hrs, respectively. Blood samples are centrifuged at 2000 rpm for 10 mins (Remi Equipment, Mumbai, India) and drug concentration after deproteinization with mobile phase was determined by HPLC assay (Shimadzu, Kyoto Corporation, Japan). Result was shown in Table 6.

#### 3.7.5. Procedure for Separation of Plasma [19]

Firstly, 10 mL iodine-free saline solution and 1 mL of heparin were taken into a beaker to wash the syringe properly. Then blood samples were taken from the marginal ear vein of the rabbits. The blood sample was then subjected to cooling centrifuge along with 5% TCA solution (anticoagulating agent). The cooling centrifuge was operated for 10 min and then it was passed through the column of silica gel to absorb the impurities.

#### 3.7.6. HPLC Assay [20]

The determination of drug in plasma was performed by HPLC assay using methanol and phosphate buffers, pH 4.3 (75 : 25 vol*\*vol) mixtures as mobile phase delivered at a rate of 1.0 mL*\*min by HPLC pump (LC-20 AT) and the detector (SPD-20A). Twenty microliters of injected volume was eluted in column at room temperature. The column eluent was monitored at 236 nm.

## 4. Results and Discussion

w*\*o emulsion solvent evaporation technique was used to prepare the microsphere of MFH using different polymers. Formulations F1–F8 were prepared by using drug with different ratios of pectin (1 : .5, 1 : 1, 1 : 1.5, 1 : 2, 1 : 2.5, 1 : 3, 1 : 3.5, and 1 : 4) and formulations F9–F12 were prepared by using drug with different polymers in the same ratio (1 : 1) for development study which was shown in Table 1. Evaluation of all formulations like percentage yield, drug content estimation, drug entrapment, percentage of moisture loss, size distribution, morphological study by scanning electron microscopy (SEM), drug polymer interaction, *in vitro* drug release, and *in vivo* studies were done accordingly. The percentage yield of all the formulations was found to be satisfactory as shown in Table 2. It can be due to the involvement of process parameters. Drug entrapment efficiency (DEE) of F2 formulation found to be high because the drug is fully dispersed in the polymer phase by continuous stirring for a period of 5-6 hrs. Very less amount of moisture was loss after 24 hrs except F3, indicating negligible presence of water in all the formulations as shown in Table 2.Table 2 showed the particle size of all the formulations found to be satisfactory and within the range of (34.56 to 83.66 *μ*m). This narrow range of particle size can be attributed as the effect of stirring time and stirring speed during preparation of microspheres. The microspheres obtained under these conditions were mostly spherical and without aggregation. To detect the surface morphology of microspheres, scanning electron microphotographs (SEM) study at different magnifications were done as shown in Figure 10 at 100x and Figure 11 at 500x, respectively.

**Figure 1:**
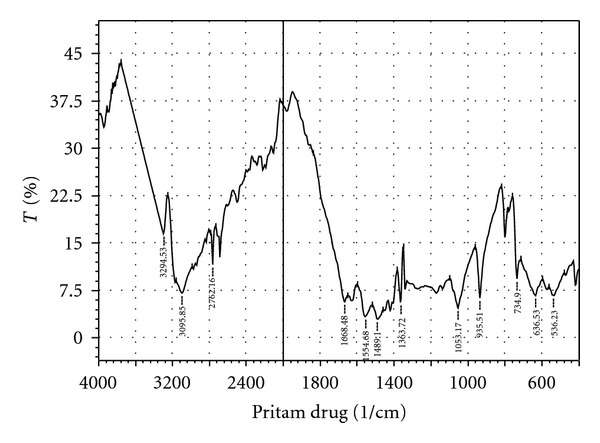
FT-IR spectrum of Metformin HCl.

**Figure 2:**
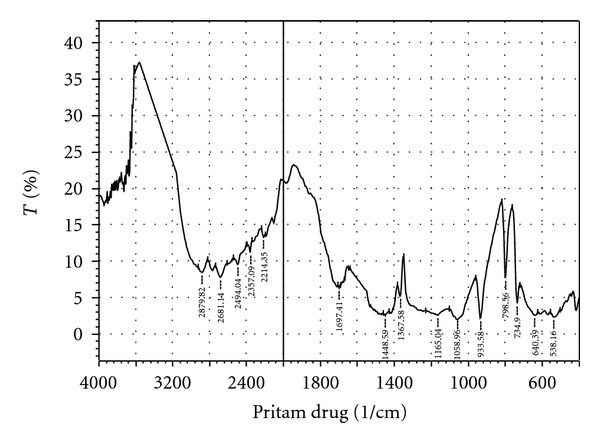
FT-IR spectrum of pectin with Metformin HCl.

From the infrared spectra [21, 22] Figures 1 and 2, it is clearly evident that there were no interactions of the drug with the polymer. The main peak in the spectrum of the drug metformin hydrochloride, both free and with polymer, does not show any substantial difference. The IR spectra show a peak at 1688.48, which signifies the presence of C=N (stretch) functional group. At 1254.14, a peak is observed which signifies the presence of C–N stretching. At 1473.34 wave numbers, a peak is also observed which signifies the presence of C–H (bend in plane). Simultaneously, a peak at 732.97 wave numbers signifies the presence of N–H (rocking) functional group. All the peaks were observed at the finger print region of the FT-IR spectra. This proves the fact that there is no potential incompatibility of the drug with the polymer (pectin) used in the formulations. Hence, the formula for preparing metformin hydrochloride microsphere with pectin can be reproducing in an industrial scale without any apprehension of possible drug-polymer interactions. After UV scanning, it was found that at 233 nm the drug-polymer solution give the highest absorbance which is similar to the absorbance given by the pure drug at UV region.

**Figure 3:**
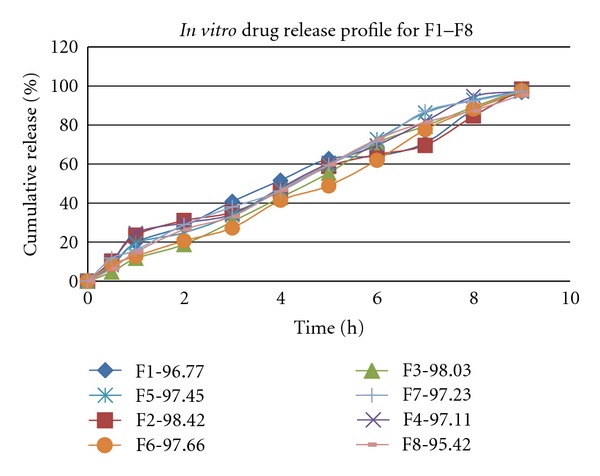
Comparative cumulative amount of MFH released from microsphere prepared by pectin in different ratios.

**Figure 4:**
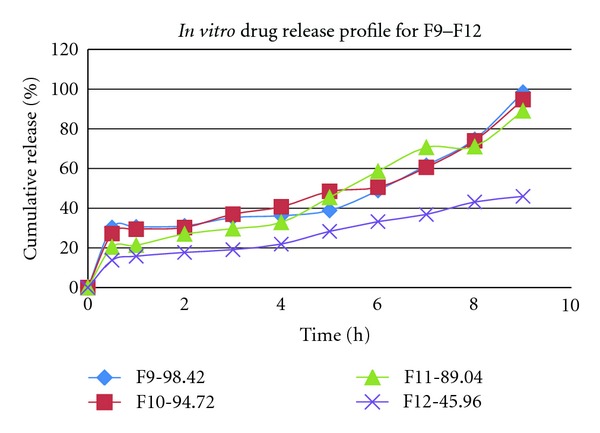
Comparative cumulative amount of MFH released from microsphere prepared by pectin (F9), ethyl cellulose (F10), HPMC (F11), and Acrycoat S100 (F12) in same (1 : 1) ratio.

The formulations F2 and F3 show a constant and high release in the dissolution profile, so among from these two, F2 was taken for development study, due to its better result compared in other evaluation parameter. For further development of pectin microsphere, F9–F12 formulations were prepared by using different polymers in the same ratio (1 : 1) by solvent evaporation method. In case of pectin formulation (F9), the drug release was 98.42% up to 9 hrs. Comparatively at the same ratio ethyl cellulose (F10) given 94.72%, HPMC (F11) given 89.04% and Acrycoat S100 (F12) given 45.96% up to 9 hrs, respectively. So we can conclude that natural pectin gives better controlled release compared to other polymer. The *in vitro* drug release data were summarized in Figure 3 for F1–F8 and Figure 4 for F9–F12, respectively. All the formulations considered for development were found to release MFH in a controlled manner. To describe the kinetics of drug release from microspheres, release data was analyzed according to different kinetic equations described in text in Table 3. Release data of F9, F10, F11, and F12 obeys zero order kinetic as well as Higuchi's square root equations. The formulation F9 prepared by w*\*o emulsion solvent evaporation method was found the release maximum in phosphate buffer pH 6.8. Putting all the data in different release kinetic models and comparing the coefficient of regression (*r* ^2^), it was found that F9, F11, and F12 tend to fit with the Fickian diffusion model given by Higuchi confirming drug release by diffusion mechanism, whereas F9, F10, F11 and F12 fits with zero order kinetic model.

**Figure 5:**
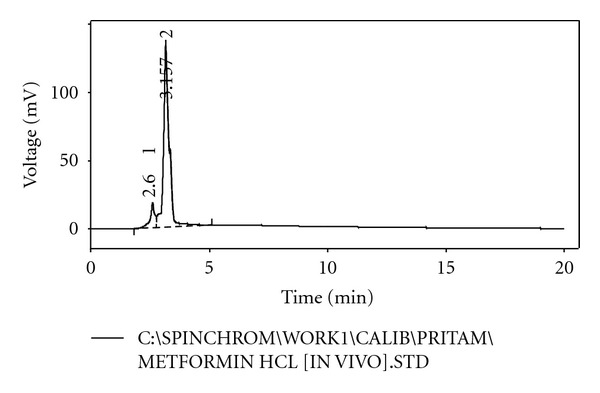
HPLC chromatogram of pure metformin hydrochloride.

**Figure 6:**
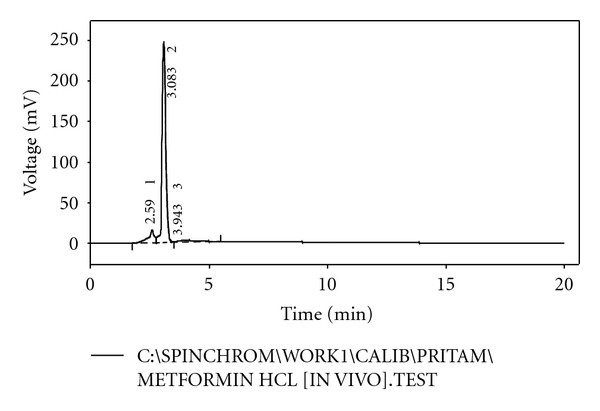
HPLC chromatogram of formulated sample (F9).

**Figure 7:**
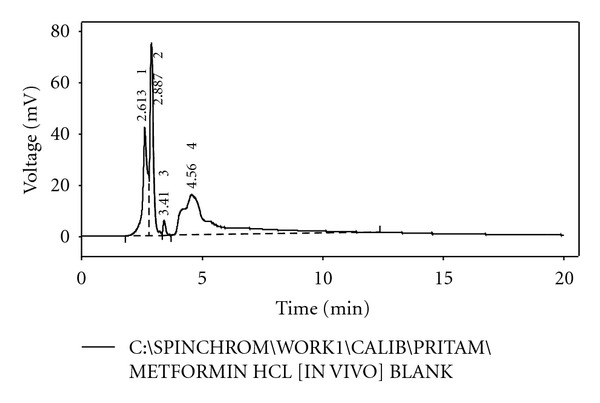
HPLC chromatogram of blank sample.

**Figure 8:**
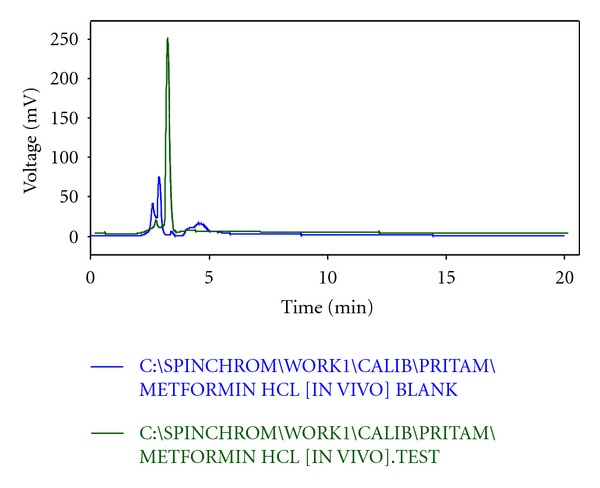
Overlap of HPLC chromatogram between blank and test.

**Figure 9:**
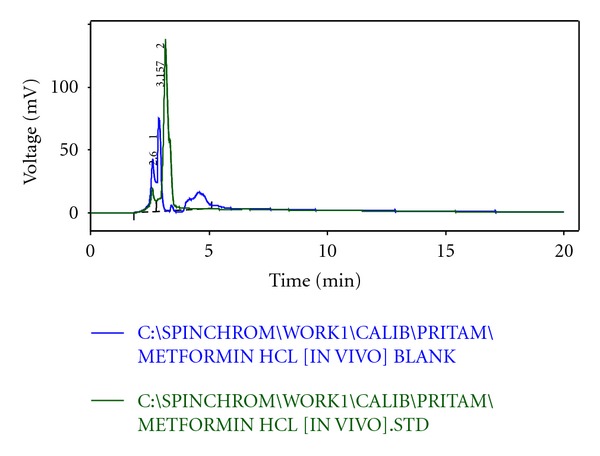
Overlap of HPLC chromatogram between blank and standard.

**Figure 10:**
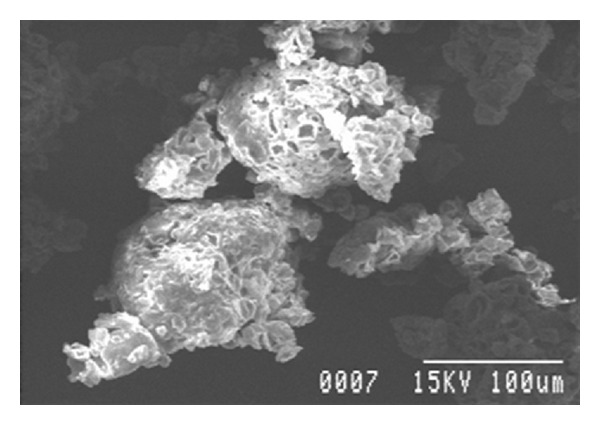
SEM photograph of microspheres (Magnification ×100).

**Figure 11:**
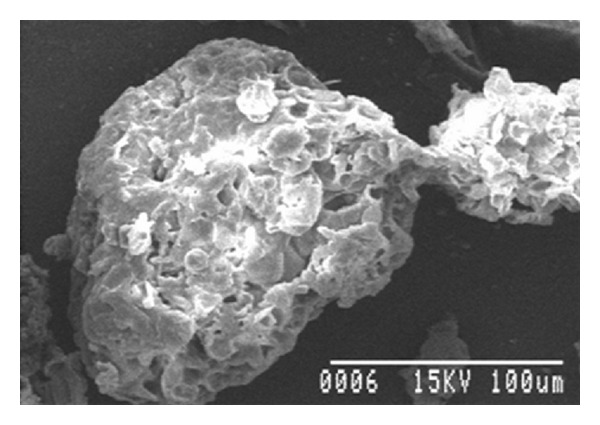
SEM photograph of microspheres (Magnification ×500).

From the HPLC determinations, it has been found that the test samples and the standard sample do not show any significant fluctuation in relation to their retention time (Figures 5, 6, 7, 8, and 9). Thus, it can be inferred that the test sample (F9) showed significant release and presence of drug within the plasma (Tables 5 and 6). In the microsphere-treated rat, the blood glucose level was steadily decreased from 1st to 5 hr and it was 206.1 to 297.5. It was concluded that the effect of antidiabetic activity of microspheres was in sustained action. Values are expressed as mean ± SEM and statistically significant when compared with diabetic controls (*P* < 0.05) (Table 4).

## 5. Conclusion

The experimental design supported product development and development procedure yielded the desired microspheres with drug release for extended period of time. The polymer combination of Pectin, Ethyl Cellulose, HPMC and Acrycoat S-100 as release retardant, resulted, microspheres with good yield and moderate entrapment. The optimized pectin microsphere of MFH is expected to provide the clinicians with a new choice of an economical, safe and more bioavailable formulation in the management of type II diabetes mellitus. Therefore, it may be concluded that drug loaded pectin microspheres will be a suitable delivery system for metformin hydrochloride, and may be used for effective management of NIDDM.

Pectin was found to be a suitable alternative of semi synthetic polymers, which can be further employed in an industrial scale as an efficient release retardant in the formulation of microspheres.

## Figures and Tables

**Table 1:** Formulation development design.

Formulation	Drug : polymer	Polymers used	Method of preparation
F9	1 : 1	Pectin	W*\*O emulsion solvent evaporation
F10	1 : 1	Ethyl cellulose	Solvent evaporation
F11	1 : 1	HPMC	Solvent evaporation
F12	1 : 1	Acrycoat S100	Solvent evaporation

**Table 2:** Physicochemical parameters of microspheres.

Formulation	Yield (%) (X ± SD)	Actual drug content (mg) (X ± SD)	Theoretical drug content (mg)	Drug entrapment efficiency (%) (X ± SD)	Particle size (*μ*m) (X ± SD)	Moisture loss (%)
F1	85.2 ± .098	17.98 ± .116	66.66	26.97 ± .167	34.56 ± .213	1.07
F2	84.00 ± .078	33.67 ± .014	50.02	67.34 ± .178	38.43 ± .265	1.08
F3	81.76 ± .105	22.43 ± .018	40.21	55.78 ± .161	58.59 ± 178	2.25
F4	84.8 ± .142	17.79 ± .011	33.33	53.37 ± .132	49.67 ± .189	1.13
F5	78.8 ± .112	13.81 ± .078	28.57	48.33 ± .096	83.66 ± .208	1.22
F6	84.8 ± .069	18.27 ± .063	28.21	64.76 ± .189	56.64 ± .228	1.49
F7	88.08 ± .117	15.37 ± .035	25.22	60.94 ± .154	69.22 ± .256	1.43
F8	86.68 ± .098	15.69 ± .021	26.07	60.18 ± .089	71.34 ± .159	1.55
F9	84.00 ± .078	39.67 ± .014	50.02	67.34 ± .178	34.56 ± .213	1.07
F10	86.90 ± .035	20.80 ± .032	50.00	41.60 ± .153	39.04 ± .194	0.89
F11	76.10 ± .161	14.91 ± .063	51.09	29.82 ± .119	42.31 ± .231	1.12
F12	80.50 ± .073	16.34 ± .124	50.00	32.68 ± .096	48.21 ± .176	1.06

Value represent mean ± SD (*n* = 3).

**Table 3:** *In vitro* drug release kinetics.

Serial number	*r* ^2^ value		
Formulation code	Zero order	First order	Higuchi
F9	**0.926**	0.769	0.942
F10	**0.946**	0.761	0.856
F11	**0.947**	0.912	0.931
F12	**0.973**	0.949	0.927

**Table 4:** Effect of pectin microspheres of metformin HCl on alloxan induced diabetic rats.

Serial number	Design of control	Drug	Blood glucose level mg/dL			
			0 hr	1 hr	3 hr	5 hr
1	Diabetic control	—	304.7 ± 5.2	312.3 ± 1.4	301.8 ± 3.5	318.2 ± 7.1
2	Placebo	—	101.2 ± 9.6	134.7 ± 7.1	137.7 ± 5.8	131.9 ± 5.8
3	Pure metformin HCl	25 mg/kg	298.7 ± 6.4	134.4 ± 5.3^∗^	156.2 ± 7.8^∗^	205.3 ± 7.8^∗^
4	Pectin microsphere	25 mg/kg	297.5 ± 13.6	206.1 ± 4.3^∗^	192.5 ± 5.4^∗^	173.1 ± 9.3^∗^

*N* = 6, ^∗^ *P* < 0.001 versus control, values are expressed as mean ± SEM. ^∗^Statistically significant when compared with diabetic controls (*P* < 0.05).

**Table 5:** HPLC observation of standard sample.

Serial number	Retention time (min)	Area (mV·s)	Height (mV)	Area (%)	Height (%)	WO5 (min)
1	2.600	252.728	18.832	10.2	12.1	0.18
2	3.157	2236.742	137.046	89.8	87.9	0.20

	Total	2489.470	155.877	100.0	100.0	

**Table 6:** HPLC observation of test sample with standard.

Serial number	Retention time (min)	Area (mV·s)	Height (mV)	Area (%)	Height (%)	WO5 (min)
01	2.590	302.087	16.545	9.9	6.2	0.19
02	3.083	2609.617	247.284	85.5	92.8	0.16
03	3.943	142.206	2.523	4.7	0.9	1.37

	Total	3053.911	266.352	100.0	100.0	

Retention time of test: 2.590 min; retention time of standard: 2.600 min.
